# Correlated Biogeographic Variation of Magnesium across Trophic Levels in a Terrestrial Food Chain

**DOI:** 10.1371/journal.pone.0078444

**Published:** 2013-11-04

**Authors:** Xiao Sun, Adam D. Kay, Hongzhang Kang, Gaston E. Small, Guofang Liu, Xuan Zhou, Shan Yin, Chunjiang Liu

**Affiliations:** 1 School of Agriculture and Biology, Shanghai Jiao Tong University, Shanghai, China; 2 Department of Biology, University of St. Thomas, St. Paul, Minnesota, United States of America; 3 State Key Laboratory of Vegetation and Environmental Change, Institute of Botany, Chinese Academy of Sciences, Beijing, China; 4 Shanghai Jiao Tong University Research Centre for Low Carbon Agriculture, Shanghai, China; Scottish Association for Marine Science, United Kingdom

## Abstract

Using samples from eastern China (c. 25 – 41° N and 99 – 123° E) and from a common garden experiment, we investigate how Mg concentration varies with climate across multiple trophic levels. In soils, plant tissue (Oriental oak leaves and acorns), and a specialist acorn predator (the weevil *Curculio davidi*), Mg concentration increased significantly with different slopes from south to north, and generally decreased with both mean annual temperature (MAT) and precipitation (MAP). In addition, soil, leaf, acorn and weevil Mg showed different strengths of association and sensitivity with climatic factors, suggesting that distinct mechanisms may drive patterns of Mg variation at different trophic levels. Our findings provide a first step toward determining whether anticipated changes in temperature and precipitation due to climate change will have important consequences for the bioavailability and distribution of Mg in food chain.

## Introduction

Magnesium (Mg) likely has important influences on terrestrial ecosystems because of its critical role in many physiological processes [1]. Magnesium is the most abundant divalent cation in living cells; it is essential for maintaining DNA and RNA structure, for stabilizing cell membranes and walls, and for catalyzing many condensation and hydrolysis reactions [2]. In plants, Mg is necessary for photosynthesis because it is the central element of chlorophyll and is a cofactor of a series of enzymes involved in carbon fixation [3]. In animals, Mg is involved in regulating body temperature [4], [5], [6], and Mg deficiency is known to cause a severe metabolic disease, grass tetany, in ruminants [7]. At the same time, Mg availability likely varies across abiotic gradients. It may vary with precipitation because Mg has a high hydrated radius that sorbs less to colloids than other cations [8], making it highly prone to leaching. It may also vary with temperature, which generally affects nutrient mineralization rates [9]. Additionally, climatic factors, especially temperature, can directly affect Mg concentration in organisms by influencing virtually all physiological rate processes and demands [10], [11]. Understanding the relationships between Mg in plants and consumers across abiotic gradients should thus provide important new information about physiological and ecological ramifications of mineral nutrients in terrestrial ecosystems.

Despite the likely importance of Mg and other minerals in terrestrial ecosystems, little is known about patterns and causes of geographic variation for most mineral nutrients [12]. Latitudinal variation in plant carbon (C), nitrogen (N), and phosphorus (P) concentrations and stoichiometries have been well documented, and are hypothesized to be caused by trends in temperature and/or soil age[11], [13]. However, relative to patterns for C, N, and P, geographic variation of other elements in plants has received little attention [12], [14], [15]. Toward addressing that knowledge gap, Han et al. (2011) compared concentrations of 11 minerals including Mg among 1900 plant species across China. They found that plant element concentrations varied with latitude and longitude, generally varied more with precipitation than with temperature, and showed greater sensitivity to abiotic gradients when elements were environmentally limited and at relatively high concentrations in plant tissue [12]. Han et al. (2011) is an important step toward developing biogeochemical models that incorporate interactions among essential elements in living systems [12].

Less is known about geographic variation in animal elemental concentration [16]. One driver of macro-elemental variation in animals may be differential allocation to P-rich ribosomal RNA needed for rapid growth (the Growth Rate Hypothesis, Elser et al. 2003) [17]. Few studies on animals have linked variation in micro-nutrient concentration to functional traits [16], and we know of no comparison of animal Mg or other micro-minerals across a broad geographic area.

Here we extend the research of Han et al. (2011) by examining patterns of within-species variation of Mg, and by exploring how this variation ramifies upwards through the food chain [12]. We focus on Mg concentration in the Oriental oak (*Quercus variabilis*) and its common seed predator, the acorn weevil (*Curculio davidi*). *Quercus variabilis* is native to a large area in eastern Asia including mainland China, Taiwan Island, Zhoushan Islands, Korea and Japan. As a deciduous broadleaf tree, Oriental oak grows extensively in eastern China from temperate regions in the north to subtropical regions in the south (24 to 42° N in latitude and 96 to 140° E in longitude). Within its distribution, mean annual temperature (MAT) ranges from 7.2 to 23.6°C, and mean annual precipitation (MAP) ranges from 411 to 2000 mm [18], with both MAT and MAP inversely related to latitude. The acorn weevil *C*. *davidi* is a holometabolous phytophagous insect that is distributed widely with its host. The wide distribution of this specialized host-herbivore pairing gives us a rare opportunity to investigate relationships between climatic factors and mineral stoichiometry in a terrestrial food chain.

Our data include samples from across a wide latitude and climate range, and from a common garden experiment. By documenting patterns of Mg variation and climate correlates, our study suggests hypotheses about how Mg-related processes will affect food chain responses to climate change. To our knowledge, this is the first study to examine variation in a micronutrient in soils, plants, and an associated herbivore across a broad geographic area.

## Materials and Methods

### Study area

**Figure 1:**
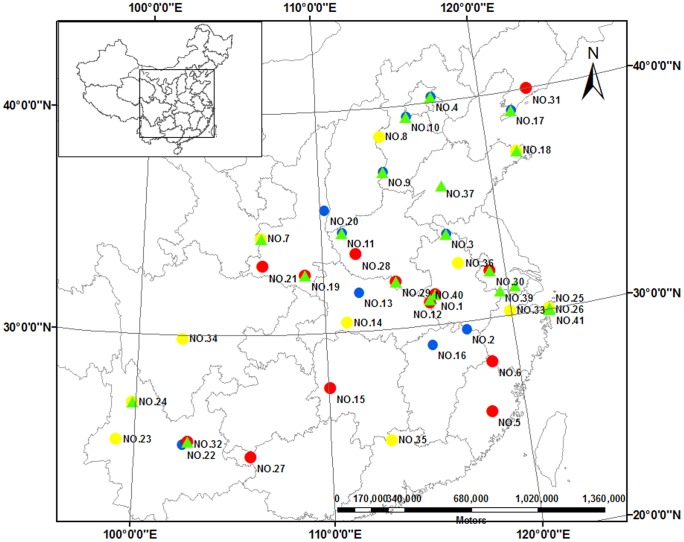
Distribution map of *Quercus variabilis* stands sampled across eastern China. The number of location can be found in the supplementary materials (Table S1). Solid circles represent soil and leaf sampling sites (yellow circles sampled in 2009, red circles sampled in 2007–2009, and blue circles sampled in 2008–2009), and the green solid triangles represent acorn and weevil larva sampling sites in 2009.

We selected sampling stands (36 stands for sampling soil and oak leaves, and 21 for acorns and weevil larvae) based on Wang et al. (1985) [19], local forestry authorities, and field trips (Fig. 1; Table S1). The study area stretches from the temperate region in northeastern and northern China to the subtropical region of central and southern China (from 24 to 41° N in latitude and from 99 to 123° E in longitude). Mean annual temperature across the area ranges from 7.8 to 20.5°C and mean annual precipitation ranges from 511 to 2029 mm. In the northern part of study area (Liaoning, Beijing, and Hebei Provinces), the zonal vegetation is temperate deciduous broadleaf forests dominated by Oriental oak, and the typical soil type is cinnamonic. In the central part of the study area (Henan, Hubei, and Jiangsu Provinces) where the climate transitions from warm-temperate to subtropical, the climax vegetation is mixed-deciduous and evergreen broadleaf forests, and the soil is characterized by brown clay layer deposition (brown soil in the Chinese soil classification system). In the southern part of the study area (Jiangxi, Guangxi, and Fujian Provinces), the zonal vegetation is typical subtropical evergreen broadleaf forest, and the typical soil is aluminum-rich acid soil (red soil in the Chinese soil classification system). All the selected stands were secondary forests with no direct harvesting, burning, or other such activities over the last three decades.

We also conducted a common garden experiment to determine the extent to which oak population differences in Mg concentration are maintained in common abiotic conditions. To do this, we chosen acorns from 11 sampling stands from our field sample along latitudinal gradients, and planted them in a greenhouse at Shanghai Jiao Tong University in December 2008. At the beginning of spring 2009, we transplanted seedlings into a 50 m ×50 m plot in an experimental field at Shanghai Jiao Tong University; seedlings were randomly interspersed with 1-m spacing between individuals.

### Sample collection

We collected leaf samples from early August to early September in 2007 – 2009. In each year, we started leaf sampling in the northern temperate region, and moved towards the southern subtropical region to sample at similar seasonal phenological stages across regions. At each site, we delimited a 20 m ×20 m plot in the middle of a south-facing slope, and five dominant trees were selected within each plot as sample trees. We selected 8 – 10 small branches in the middle of south-facing crown of each sample tree, and picked the fully developed and healthy leaves from the middle branch. We used this consistent method of collection to minimize possible developmental differences in leaf growth among sites. To obtain representative soil samples, we divided each plot into 5 sub-plots and sampled from the top layer (0–20 cm in depth) at 5 locations systematically distributed within each sub-plot. Samples were mixed within each sub-plot to make one bulk sample per plot (*n* = 5 per site).

We harvested acorns from the ground at the end of September to early November in 2009. At each site, approximately 10 kg of acorns were harvested from the plot where we collected leaf samples. We pooled all samples within a site for bulk sample preparation, as our trial test showed limited within-site variation in acorn chemical composition (Sun and Liu unpublished data). We used ∼50 acorns without weevil damage from each site to measure Mg concentration (damaged acorns have obvious scars from weevil entry and exit), and stored the rest at room temperature for 1 – 5 days for incubating weevil larvae. This species of weevil is endoparasitic and develops within a single host acorn before emerging as a mature larva. We collected larvae within 12 h of emergence from acorns.

We collected leaf samples from the common garden experiment in 2011, when oaks were three years old. For each of the 11 stands, we picked leaves in full sunlight from each of 20 trees. From each tree, we sampled 2 – 3 pairs of mid-stem mature leaves from 2 randomly selected branches.

### Sample preparation and chemical analyses

We placed soil samples in a shaded and ventilated environment for about one month to air dry them to constant mass. We removed acorn coats; embryos and extra-embryonic tissues were combined and are hereafter referred to as EETs. All organismal samples were cleaned with distilled water. Leaves, acorn and weevils were oven-dried for one week to constant mass (65°C for leaf and acorn, and 50°C for weevils). Soil, leaf and acorn samples were ground in 20-mesh Wiley Mill. Weevil larvae were processed using a ball mill for 1 min to completely homogenize samples. All the samples were stored in a silica gel bag, and kept cool and dry until chemical analysis.

Magnesium concentration in all samples was determined with inductively coupled plasma atomic emission-spectrometry (ICP). The standard BCR-60 Lagarosiphon Major by the Community Bureau of Reference (BCR, Belgium) was used as reference material for spot-checks during the analysis. For analysis, the mass of samples was obtained to the nearest 0.1 mg, digested with nitric acid (trace-metal grade pure), and then diluted in 100 ml of distilled water.

### Climate and geographical position data collection

Global positioning system (Thales USA) was used to determine geographical position and altitude (m a.s.l). Mean annual temperature (MAT, °C), mean annual precipitation (MAP, mm), average diurnal range of temperature (DRT, °C), and annual precipitation seasonality (APS, %; coefficient of variation of monthly mean precipitations) were estimated using the global climate dataset with a resolution of 0.0083×0.0083 (ca. 1 km ×1 km) obtained from http://www.worldclim.org/. Growing season length (GSL, days) was estimated with records from 756 climatic stations in China using spatial interpolation method (during 1954 – 2007).

### Statistical analysis

All elemental concentrations were log_10_-transformed before analyses to improve data normality. Linear regression was employed to fit the relationships between Mg concentration and climatic factors and latitude. Slopes of soil and organism Mg in relation to environmental factors were calculated using bivariate Reduced Major Axis regression (RMA) models. Reduced Major Axis regressions were performed using the program RMA [20] (http://www.bio.sdsu.edu/pub/andy/RMA.html). Slopes of relationships between soil or organism Mg concentration and environmental factors were compared using covariance analysis.

Multicollinearity among explanatory variables may result in the exclusion of ecologically more causal variables from multiple regression models [21]. To overcome the problems caused by collinearity in this study, we used hierarchical partitioning. This method can provide more insight into organism stoichiometry-environment relationships than traditional regression methods by decomposing the variation in response variable(s) into independent components which reflect the relative importance of individual predictors or groups of predictors and their joint effects [22]. Hierarchical partitioning was conducted using the ‘hier.part package’ version 0.5 – 1 [23], part of the R statistical package. To further examine the extent to which climate impacts on organism Mg are mediated through soil and food, path analysis was performed to partition direct effects of climate on oak and weevil Mg and indirect effects of resource transfer through the food chain [24]. In addition, possible effects of soil Mg on leaf Mg, and of acorn Mg on weevil Mg were examined using Spearman's rank correlation. All statistical and regression analyses were conducted using SPSS (version 19.0, IBM Corp., Somers, NY, USA) and R 2.2.1 (R Development Core Team 2005).

## Results

### Latitudinal patterns of soil and organism Mg variation

**Figure 2:**
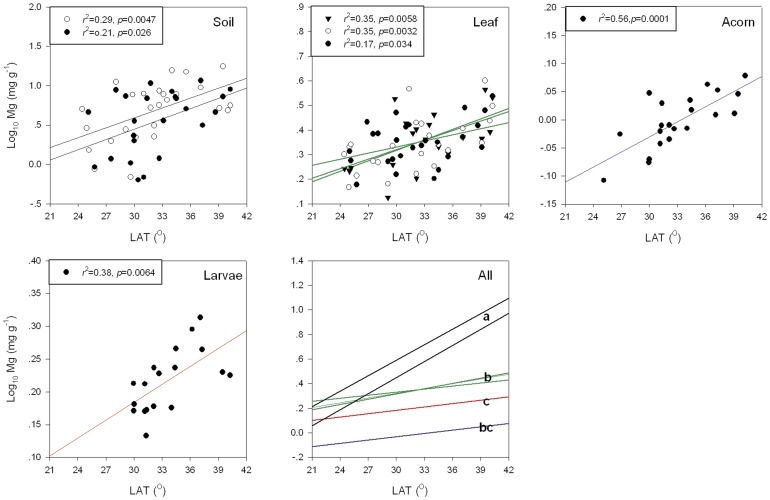
Relationships between Mg concentration in soil, Oriental oak leaves and acorns, or larvae of the weevil *Curculio davidi* and latitude. Sampling was carried out across eastern China. Reduced Major Axis (RMA) regression slopes are 5.74 for soil, 1.63 for leaf, 0.91 for acorn and 1.11 for weevil larvae. Solid triangles, open circles, and solid circles are for data from 2007, 2008, and 2009, respectively. Different letters in the panel “All” indicate significant differences between slopes in a post-hoc comparison. The data of weevil Mg concentration (2.52 mg g^-1^) from Anning and Lijiang (2.10 mg g^-1^), Kunming, were not included in the statistics.

On average, Mg concentration was highest in soils, followed by leaves, weevil larvae, and acorns (Table S2). Across the latitudinal gradient, we observed 18-fold variation in soil Mg that was reflected in roughly 2-fold variation in oak leaves, acorns, and weevil larvae (Table S2). Both soil and organism Mg concentration showed significant latitudinal trends, increasing from south to north. However, the difference between soil Mg and organism Mg increased at higher latitudes because the positive relationship between Mg concentration and latitude was steeper for soils than it was for leaves, weevils, or acorns (Fig. 2; Table S3).

Leaf Mg in the common garden experiment varied much less (assessed as the coefficient of variation (CV), CV = 11%) among trees than did leaf Mg in samples collected in the geographic survey (CV = 29%) (Table S4), and there was actually an almost-significant decrease in leaf Mg concentration with latitude of seed origin (*p* = 0.059; Fig. S1).

### Climatic influence on soil and organism Mg concentration

Magnesium in soil, oak leaves and acorns, and weevil larvae were correlated with most of the five climatic factors included in our analysis (Table 1). Most notably, soil Mg was closely (negatively) associated with diurnal range of temperature (DRT) and mean annual precipitation (MAP), acorn Mg was (negatively) associated with growing season length (GSL), and weevil larvae Mg was (negatively) correlated with mean annual temperature (MAT) and DRT (Table 1). Spearmańs rank correlations showed that although resource-organism (i.e., soil-oak and acorn-weevil) Mg concentrations were positively related (Table 2), variation in organism Mg was generally more closely associated with climatic factors than with resource Mg (Table 1).

**Table 1:** Results of hierarchical partitioning for the effects of climatic factors on soil Mg, and the effects of climatic factors and Mg available on leaf, acorn and weevil larva Mg.

	Full Model (r^2^)	Contribution of the individual predictor (%)							
		MAT	DRT	MAP	ASP	GSL	Soil	Acorn	n
Soil	0.53	6.81	36.73	32.64	18.73	5.1	-	-	35
Leaf	0.31	15.6	8.73	19.93	32.06	15.18	8.5	-	36
Acorn	0.6	14.98	4.46	11.05	10.97	51.78	6.77	-	21
Weevil	0.54	45.03	21.48	7.2	12.34	11.19	-	2.77	20

Note: see Full Methods for the abbreviations.

**Table 2:** Spearmańs rank correlations (ρ) between leaf and soil Mg, acorn and soil Mg, and weevil and acorn Mg. Mg (mg g^-1^) is log_10_-transformed before analysis.

	ρ	*n*	*p*
Leaf *vs*. soil	0.396	34	0.0205
Acorn *vs*. soil	0.152	21	0.505
Weevil *vs*. acorn	0.435	19	0.0614

**Figure 3:**
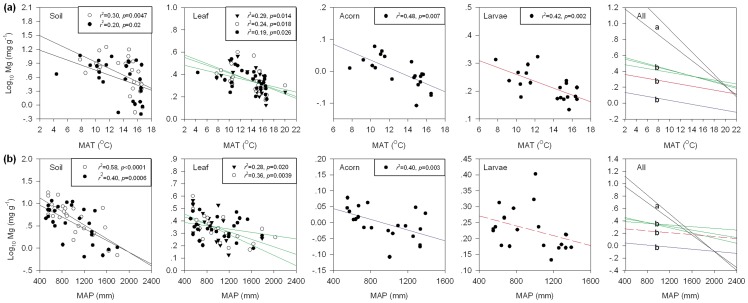
Associations between soil, Oriental oak leaf and acorn, and weevil larva Mg concentrations and mean annual temperature (MAT) and mean annual precipitation (MAP) across temperate-subtropical biomes in eastern China. Reduced Major Axis (RMA) regression slopes are −2.74 for soil, −0.82 for leaf, −0.54 for acorn and −0.57 for weevil larva against MAT, and for MAP, slopes are −2.44 for soil, −0.65 for leaf, −0.34 for acorn and −0.36 for weevil larva. Solid triangles, open circles, and solid circles are for data from 2007, 2008, and 2009, respectively. Broken line indicates no significance. Different letters in the panel “All” indicate significant differences between slopes in a post-hoc comparison.

Because of the close relationships and multicollinearity among the five climatic factors included in the study (Table S5, see also Han et al. 2011) [12], we focused the bulk of our analysis on temperature (MAT) and precipitation (MAP), the abiotic factors that explain most geographic variation in plant composition and structure [11], [12]. Soils and organism tissue generally had lower Mg concentration in warmer, wetter areas, but MAT and MAP were both more strongly associated with variation in soil than with variation in organism Mg (Fig. 3). Surprisingly, relationships of MAT and MAP to acorn Mg had similar slopes to MAT and MAP relationships with weevil Mg (Fig. 3). Soil Mg was more strongly associated with MAP than with MAT, while acorn and weevil Mg showed a stronger associated with MAT than with MAP (Table S6).

Path analysis showed that associations between soil and organism Mg and other climatic factors were driven by intercorrelations among explanatory variables, and the direct effects of MAT and MAP on organism Mg concentration were larger than the indirect effects through affecting the resource Mg; for instance, the effect of MAT on leaf Mg was composed of a direct effect (−0.31) and an indirect effect on soil Mg (0.04) (Fig. S2).

Results from the common garden experiment showed no significant differences in leaf Mg concentration with site-of-origin MAT and MAP (Fig. S1).

## Discussion

Magnesium is a component of key physiological processes in plants and animals, but little is known about how its distribution in food chain varies across climatic gradients. Here we show that Mg concentration in soil and in the leaves and fruits of a dominant tree (the Oriental oak) varies with abiotic factors across a broad latitude range in China. In addition, we also show that similar variation exists in larvae of a seed predator of Oriental oak, the weevil (*C. davidi*), suggesting that climate-correlated variation in mineral element availability can cascade upward to higher trophic levels. To our knowledge, our study is the first to document plant and associated-herbivore variation in mineral nutrient concentration across natural abiotic gradients.

### Variation of organism Mg along latitudinal gradients and trophic levels

One of our findings is the substantial intraspecific variation in plant and insect Mg concentration across a geographic region. We documented a much larger range (1.33 – 5.41 mg g^-1^) of leaf Mg concentration in Oriental oak forests in our samples (Table S2) than in a similar intraspecific comparison in Spain (1.1 – 1.7 mg g^-1^) [25]; our range in leaf Mg concentration was even larger than one across 1900 species from China (1.65 – 3.75 mg g^-1^) [12]. Our values are still, however, within the putative optimal range for plants [26]. In contrast to the extensive geographic variation, leaf Mg varied only slightly among seedlings from different regions planted in a common garden, suggesting that latitudinal variation in leaf Mg concentration is driven mainly by phenotypically plastic responses to environmental factors. We also present one of only a few data sets (e.g., De Frenne et al. 2011) showing geographic variation in fruit micronutrient concentration (Fig. 2) [27]. Our results indicate that fruit Mg concentration variation can be substantial (range in acorns  = 0.78 to 1.2 mg g^-1^), although narrower than that in leaves (Table S2). The nearly two-fold variation for Mg concentration 1.36 to 2.53 mg g^-1^ in weevil larvae was surprising given the putative importance of elemental homeostasis in consumer taxa [16], [28]. We did find some evidence of organism Mg homeostasis, as Mg concentration in leaves, acorns and weevil larvae all varied less with latitude than did soil Mg (Fig. 2). However, slopes of the relationships to latitude did not differ significantly between acorns and weevil larvae, and the relationship between acorn Mg and weevil Mg was almost significant (*p* = 0.06). Together, these results suggest that consumer Mg concentration may track resource Mg concentration in this system; similar associations between herbivore and resource composition have been documented for phosphorus concentration [29].

Across all samples, Mg concentration increased from acorns < acorn predators (weevil larvae) < oak leaves < soils. These results suggest that soil Mg concentration is likely not limiting biomass production in this system and is not accumulating at higher trophic levels. One caveat, however, is that we measured total soil Mg concentration, rather than Mg availability for Oriental oaks. Regardless, our results suggest that future research should focus on potential costs to plants and herbivores of exposure to high Mg soils.

### Potential causes of Mg variation across temperate-subtropical biomes

Our results document geographic variation in Mg concentration and abiotic correlates. Below we speculate on possible causes of main patterns with the aim of motivating additional research to identify mechanisms.

We found that soil Mg concentration varied more with MAP than with MAT, possibly because the large hydrated radius of the Mg cation makes it more prone to leaching than other biologically active cations [8]. The strong association between soil Mg and precipitation is consistent with previous among- and within-species comparisons [12], [30], but see Johansson (1995) [15].

The geographical variation in organism Mg concentration that we documented is likely driven by a combination of resource availability and demands for functional performance related to climate factors. Variation in oak leaf Mg was most strongly associated with soil Mg, MAT, and APS, suggesting that leaf Mg depends largely on Mg availability and factors influencing oak growth rate. This explanation is consistent with experimental comparisons across soil fertility and plant growth rates [31], [32]. Lower leaf Mg concentration in low soil Mg environments at lower latitudes is consistent with plant acclimation to older, highly leached soils, as proposed for N and P (“the soil substrate age hypothesis”, Reich and Oleksyn 2004) [11]. Acorn Mg concentration was strongly (negatively) associated with GSL (Fig S3), and was much less variable among our samples than leaf Mg concentration, which suggest priority allocation of this mineral to reproductive tissue. One possibility for the high acorn Mg at high latitude is that Oriental oaks allocate more Mg to seeds for rapid photosynthesis by seedlings in areas with short growing seasons.

Patterns of variation in weevil larvae suggest functional demands rather than resource availability principally determine Mg concentration. We found weevil Mg was closely associated with MAT and DRT across sites (Fig S3), but was only weakly related to variation in Mg concentration in its food source, acorns. High weevil Mg concentration in high-latitude sites with short growing seasons and low temperatures may be caused by Mg sequestration to meet physiological demands of exposure to low temperatures. The importance of Mg for body temperature regulation during hibernation is well established in mammals [5], [6]; but its role in insect temperature physiology is poorly understood. Alternatively, weevil Mg associations with GSL and MAT may be driven by accumulation of Mg-free biomolecules (e.g., lipids); one possibility is that longer growing seasons and higher temperatures at southern latitudes select for greater energy storage which in turn dilutes body Mg concentration [33]. Evaluation of these hypotheses will require more comprehensive analysis of weevil concentration and performance assays to test the effects of changes in temperature, GSL and DRT on insect Mg concentration.

## Conclusions

We document three main patterns: Mg concentration varies significantly with latitude in soil and in organisms at different trophic levels; patterns of variation differ among trophic levels such that soil and organism Mg are more divergent at higher latitudes, and environmental correlates of Mg variation differ to some extent for soils, leaves, acorns, and acorn predators (weevils). Our results suggest that distinct mechanisms may drive geographical patterns of Mg variation at different trophic levels, leading in turn to different response to climate change across latitudinal gradient. These clear consistent patterns at least suggest ways in which Mg in ecosystems will vary with climate change.

Understanding variation in material composition in food chain across geographic areas should be useful for predicting climate change effects on ecosystem function. Our geographic comparison of Mg concentration suggests that warm, wet conditions will reduce imbalances between plant Mg concentration and Mg availability. A focus on Mg and other elements in organism biomass provides currencies for comparing environmental conditions to organism function that can be applied across diverse taxa [34]. By coupling the chemical properties and roles in organism with patterns of variation, these findings broaden the knowledge of mechanisms underlying organism Mg variation responds to climate change.

## Supporting Information

Figure S1 **Variation in leaf Mg concentrations (mg g^-1^) with latitude (LAT) (A), mean annual temperature (MAT, °C) (B) and mean annual precipitation (MAP, mm) (C) in 11 Oriental oak field stands (filled symbols, bold lines) and in the common garden experiment (open symbols, light lines).** Field data are from 2009. LAT, MAT, and MAP for the common garden comparisons are data from site of origin. Dashed lines indicate relationships are almost significant. For LAT, the leaf in field (*r* ^2^ = 0.48, *p* = 0.019) and in common garden (*r* ^2^ = 0.34, *p* = 0.059). For MAT, the leaf in field (*r* ^2^ = 0.30, *p* = 0.059), and for MAP, the leaf in field (*r* ^2^ = 0.64, *p* = 0.0031).
(DOCX)

Figure S2 **Path analyses of the relationships between Mg in soil (A), leaf (B), acorn (C) and weevil larva (D) and environmental factors (soil Mg or acorn Mg, MAT (mean annual temperature) and MAP (mean annual precipitation)) in Oriental oak stands across eastern China.** Numbers in bold type show the Pearson correlation coefficients among the variables, whereas numbers in parentheses partition the Pearson correlation coefficients into direct and indirect effects of environmental factors on leaf, acorn and weevil Mg (i.e. attributable to indirect relationships with the other predictor variable) based on average data collected in 2007, 2008, 2009 for soil and leaf, and data collected 2009 for acorn and weevil larva.
(DOCX)

Figure S3 **Relationships between acorn Mg content and growing season length (GSL, days) (left panel), and between weevil larva Mg content and average range of temperature (DRT, °C) (right panel) in Oriental oak stands across temperate-subtropical biomes in eastern China.**
(DOCX)

Table S1 **Summary of geographical location for sampling sites across eastern China.**
(DOCX)

Table S2 **Statistical summary of Mg concentration (mg g^-1^) in organism (leaf, acorn and weevil larva) and soil in Oriental oak stands in eastern China.**
(DOCX)

Table S3 **Summary of the covariance analysis of slopes of soil and organism (leaf, acorn and weevil larva) Mg against latitude (LAT), mean annual temperature (MAT, °C) and mean annual precipitation (MAP, mm).**
(DOCX)

Table S4 **Statistical summary of leaf Mg concentration in the field survey and common garden experiment.**
(DOCX)

Table S5 **Correlations among the five climatic variables using climate data from all sampling sites.**
(DOCX)

Table S6 **General Linear Model results of relationships between soil, leaf, acorn or weevil Mg and climate variables (MAP =  mean annual precipitation, MAT = mean annual temperature). “ns” indicates no significant relationship.**
(DOCX)
