# Supplementary material for: Correlated Biogeographic Variation of Magnesium across Trophic Levels in a Terrestrial Food Chain
Source: PLoS One. 2013 Nov 4;8(11):e78444. doi: 10.1371/journal.pone.0078444 (PMC3817214; doi:10.1371/journal.pone.0078444)
Supplement: Figure S1 — Variation in leaf Mg concentrations (mg g-1) with latitude (LAT) (A), mean annual temperature (MAT, °C) (B) and mean annual precipitation (MAP, mm) (C) in 11 Oriental oak field stands (filled symbols, bold lines) and in the common garden experiment (open symbols, light lines). Field data are from 2009. LAT, MAT, and MAP for the common garden comparisons are data from site of origin. Dashed lines indicate relationships are almost significant. For LAT, the leaf in field (r 2 = 0.48, p = 0.019) and in common garden (r 2 = 0.34, p = 0.059). For MAT, the leaf in field (r 2 = 0.30, p = 0.059), and for MAP, the leaf in field (r 2 = 0.64, p = 0.0031). (DOCX) [file pone.0078444.s001.docx]

**Fig. S1** Variation in leaf Mg concentrations (mg g^-1^) with latitude (LAT) (A), mean annual temperature (MAT, °C) (B) and mean annual precipitation (MAP, mm) (C) in 11 Oriental oak field stands (filled symbols, bold lines) and in the common garden experiment (open symbols, light lines). Field data are from 2009. LAT, MAT, and MAP for the common garden comparisons are data from site of origin. Dashed lines indicate relationships are almost significant. For LAT, the leaf in field (*r*^2^=0.48, *p*=0.019) and in common garden (*r*^2^=0.34, *p*=0.059). For MAT, the leaf in field (*r*^2^=0.30, *p*=0.059), and for MAP, the leaf in field (*r*^2^=0.64, *p*=0.0031).
