# Supplementary material for: Correlated Biogeographic Variation of Magnesium across Trophic Levels in a Terrestrial Food Chain
Source: PLoS One. 2013 Nov 4;8(11):e78444. doi: 10.1371/journal.pone.0078444 (PMC3817214; doi:10.1371/journal.pone.0078444)
Supplement: Figure S2 — Path analyses of the relationships between Mg in soil (A), leaf (B), acorn (C) and weevil larva (D) and environmental factors (soil Mg or acorn Mg, MAT (mean annual temperature) and MAP (mean annual precipitation)) in Oriental oak stands across eastern China. Numbers in bold type show the Pearson correlation coefficients among the variables, whereas numbers in parentheses partition the Pearson correlation coefficients into direct and indirect effects of environmental factors on leaf, acorn and weevil Mg (i.e. attributable to indirect relationships with the other predictor variable) based on average data collected in 2007, 2008, 2009 for soil and leaf, and data collected 2009 for acorn and weevil larva. (DOCX) [file pone.0078444.s002.docx]

**Fig. S2** Path analyses of the relationships between Mg in soil (A), leaf (B), acorn (C) and weevil larva (D) and environmental factors (soil Mg or acorn Mg, MAT (mean annual temperature) and MAP (mean annual precipitation)) in Oriental oak stands across eastern China. Numbers in bold type show the Pearson correlation coefficients among the variables, whereas numbers in parentheses partition the Pearson correlation coefficients into direct and indirect effects of environmental factors on leaf, acorn and weevil Mg (i.e. attributable to indirect relationships with the other predictor variable) based on average data collected in 2007, 2008, 2009 for soil and leaf, and data collected 2009 for acorn and weevil larva.
