# Supplementary material for: Correlated Biogeographic Variation of Magnesium across Trophic Levels in a Terrestrial Food Chain
Source: PLoS One. 2013 Nov 4;8(11):e78444. doi: 10.1371/journal.pone.0078444 (PMC3817214; doi:10.1371/journal.pone.0078444)
Supplement: Figure S3 — Relationships between acorn Mg content and growing season length (GSL, days) (left panel), and between weevil larva Mg content and average range of temperature (DRT, °C) (right panel) in Oriental oak stands across temperate-subtropical biomes in eastern China. (DOCX) [file pone.0078444.s003.docx]

**Fig. S3** Relationships between acorn Mg content and growing season length (GSL, days) (left panel), and between weevil larva Mg content and average range of temperature (DRT, °C) (right panel) in Oriental oak stands across temperate-subtropical biomes in eastern China.
