# Supplementary material for: Correlated Biogeographic Variation of Magnesium across Trophic Levels in a Terrestrial Food Chain
Source: PLoS One. 2013 Nov 4;8(11):e78444. doi: 10.1371/journal.pone.0078444 (PMC3817214; doi:10.1371/journal.pone.0078444)
Supplement: Table S1 — Summary of geographical location for sampling sites across eastern China. (DOCX) [file pone.0078444.s004.docx]

**Table S1** Summary of geographical location for sampling sites across eastern China.

| Sample stand number | Sites | Latitude  (°) | Longitude  (°) | Altitude  (m) |
| --- | --- | --- | --- | --- |
| NO. 1 | Huoshan, Anhui | 31.35 | 116.08 | 659 |
| NO. 2 | Qimen, Anhui | 29.61 | 117.54 | 459 |
| NO. 3 | Xiaoxian, Anhui | 34.02 | 117.06 | 117 |
| NO. 4 | Pinggu,Beijing | 40.25 | 117.12 | 260 |
| NO. 5 | Dehua, Fujian | 25.75 | 118.31 | 484 |
| NO. 6 | Pucheng, Fujian | 28.03 | 118.68 | 704 |
| NO. 7 | Tianshui, Gansu | 34.38 | 106.67 | 789 |
| NO. 8 | Shijiazhuang,Hebei | 38.69 | 113.81 | 1145 |
| NO. 9 | Xingtai, Heibei | 37.09 | 113.83 | 801 |
| NO. 10 | Yixian, Heibei | 39.48 | 115.48 | 516 |
| NO. 11 | Sanmenxia, Henan | 34.49 | 111.22 | 1121 |
| NO. 12 | Luotian,Hubei | 31.01 | 115.77 | 312 |
| NO. 13 | Nanzhang,Hubei | 31.75 | 111.93 | 237 |
| NO. 14 | Yichang,Hubei | 30.43 | 111.21 | 276 |
| NO. 15 | Huaihua, Hunan | 27.51 | 110.11 | 455 |
| NO. 16 | Yongxiu,Jiangxi | 29.09 | 115.62 | 360 |
| NO. 17 | Dalian, Liaoning | 39.11 | 121.80 | 180 |
| NO. 18 | Kunyushan, Shandong | 37.29 | 121.75 | 223 |
| NO. 19 | Ankang, Shanxi | 32.66 | 109.03 | 370 |
| NO. 20 | Huanglong, Shanxi | 35.53 | 110.27 | 960 |
| NO. 21 | Mianxian, Shanxi | 33.11 | 106.70 | 715 |
| NO. 22 | Anning, Yunnan | 24.98 | 102.44 | 1826 |
| NO. 23 | Baoshan, Yunnan | 25.12 | 99.15 | 1821 |
| NO. 24 | Lijiang, Yunnan | 26.87 | 99.87 | 1988 |
| NO. 25 | Dinghai, Zhejiang | 30.02 | 122.07 | 76 |
| NO. 26 | Damaodao, Zhejiang | 29.96 | 122.04 | 92 |
| NO. 27 | Tianlin,Guangxi | 24.43 | 105.93 | 696 |
| NO. 28 | Neixiang, Henan | 33.50 | 111.92 | 1112 |
| NO. 29 | Xinyang, Henan | 32.12 | 114.01 | 131 |
| NO. 30 | Jurong, Jiangsu | 32.13 | 119.20 | 160 |
| NO. 31 | Zhuanghe, Liaoning | 39.99 | 122.96 | 250 |
| NO. 32 | Kunming, Yunnan | 25.14 | 102.74 | 1955 |
| NO. 33 | Hangzhou,Zhejiang | 30.19 | 120.00 | 349 |
| NO. 34 | Luding, Sichuan | 29.83 | 102.38 | 1757 |
| NO. 35 | Nanling, Guangdong | 24.92 | 113.08 | 500 |
| NO. 36 | Fengyang, Anhui | 32.65 | 117.56 | 28 |
