# Supplementary material for: Correlated Biogeographic Variation of Magnesium across Trophic Levels in a Terrestrial Food Chain
Source: PLoS One. 2013 Nov 4;8(11):e78444. doi: 10.1371/journal.pone.0078444 (PMC3817214; doi:10.1371/journal.pone.0078444)
Supplement: Table S2 — Statistical summary of Mg concentration (mg g-1) in organism (leaf, acorn and weevil larva) and soil in Oriental oak stands in eastern China. (DOCX) [file pone.0078444.s005.docx]

**Table S2** Statistical summary of Mg concentration (mg g^-1^) in organism (leaf, acorn and weevil larva) and soil in Oriental oak stands in eastern China.

|  | Mean | Max | Min | SD | CV | n | Collection date |
| --- | --- | --- | --- | --- | --- | --- | --- |
| Soil | 6.35 | 17.61 | 0.69 | 4.35 | 69 | 24 | 2008 |
|  | 5.28 | 11.56 | 0.64 | 3.49 | 66 | 25 | 2009 |
| Live leaf | 2.36 | 3.67 | 1.33 | 0.63 | 27 | 20 | 2007 |
|  | 2.45 | 5.41 | 1.52 | 0.89 | 36 | 24 | 2008 |
|  | 2.28 | 3.45 | 1.51 | 0.49 | 21 | 25 | 2009 |
| Acorn | 1.00 | 1.2 | 0.78 | 0.11 | 11 | 21 | 2009 |
| Weevil | 1.71 | 2.53 | 1.36 | 0.28 | 16 | 20 | 2009 |

*The data of soil Mg concentration (30.24 mg g^-1^) from Anning, Kunming in 2009, were not included in the statistics, because it is outliers.
