# Supplementary material for: Correlated Biogeographic Variation of Magnesium across Trophic Levels in a Terrestrial Food Chain
Source: PLoS One. 2013 Nov 4;8(11):e78444. doi: 10.1371/journal.pone.0078444 (PMC3817214; doi:10.1371/journal.pone.0078444)
Supplement: Table S3 — Summary of the covariance analysis of slopes of soil and organism (leaf, acorn and weevil larva) Mg against latitude (LAT), mean annual temperature (MAT, °C) and mean annual precipitation (MAP, mm). (DOCX) [file pone.0078444.s006.docx]

**Table S3** Summary of the covariance analysis of slopes of soil and organism (leaf, acorn and weevil larva) Mg against latitude (LAT), mean annual temperature (MAT, °C) and mean annual precipitation (MAP, mm).

|  | LAT | | | MAT | | | MAP | | |
| --- | --- | --- | --- | --- | --- | --- | --- | --- | --- |
|  | Soil Mg | Leaf Mg | Acorn Mg | Soil Mg | Leaf Mg | Acorn Mg | Soil Mg | Leaf Mg | Acorn Mg |
| Leaf Mg | 0.015 |  |  | 0.039 |  |  | 0.022 |  |  |
|  | **6.303** |  |  | **4.447** |  |  | **5.583** |  |  |
| Acorn Mg | 0.001 | 0.034 |  | 0.046 | 0.055 |  | 0.034 | 0.272 |  |
|  | **11.465** | **4.171** |  | **4.161** | **3.86** |  | **4.689** | **1.234** |  |
| Weevil Mg | 0.045 | 0.34 | 0.29 | 0.015 | 0.145 | 0.871 | 0.023 | 0.591 | 0.58 |
|  | **4.232** | **0.929** | **1.153** | **6.282** | **2.184** | **0.027** | **5.469** | **0.292** | **0.311** |

*The bold is F, and normal font is p. p<0.05 means significant difference between slopes.
