# Supplementary material for: Correlated Biogeographic Variation of Magnesium across Trophic Levels in a Terrestrial Food Chain
Source: PLoS One. 2013 Nov 4;8(11):e78444. doi: 10.1371/journal.pone.0078444 (PMC3817214; doi:10.1371/journal.pone.0078444)
Supplement: Table S4 — Statistical summary of leaf Mg concentration in the field survey and common garden experiment. (DOCX) [file pone.0078444.s007.docx]

**Table S4** Statistical summary of leaf Mg concentration in the field survey and common garden experiment.

|  | Mean | Max | Min | SD | CV | n |
| --- | --- | --- | --- | --- | --- | --- |
| Field | 2.42 | 3.99 | 1.52 | 0.7 | 29 | 11 |
| Common Garden | 1.86 | 2.15 | 1.56 | 0.21 | 11 | 11 |
