# Supplementary material for: Correlated Biogeographic Variation of Magnesium across Trophic Levels in a Terrestrial Food Chain
Source: PLoS One. 2013 Nov 4;8(11):e78444. doi: 10.1371/journal.pone.0078444 (PMC3817214; doi:10.1371/journal.pone.0078444)
Supplement: Table S5 — Correlations among the five climatic variables using climate data from all sampling sites. (DOCX) [file pone.0078444.s008.docx]

**Table S5** Correlations among the five climatic variables using climate data from all sampling sites.

| Variable | DRT | MAP | APS | GSL |
| --- | --- | --- | --- | --- |
| MAT | -0.62 | 0.73 | -0.74 | 0.75 |
| DRT |  | -0.64 | 0.76 | -0.41 |
| MAP |  |  | -0.83 | 0.78 |
| APS |  |  |  | -0.65 |

Note: *MAT* mean annual temperature (°C), *MAP* mean annual precipitation (mm), *DRT* average diurnal range of temperature (°C), *APS* annual precipitation seasonality (%), and *GSL* Average growing season length (days).
