# Supplementary material for: Correlated Biogeographic Variation of Magnesium across Trophic Levels in a Terrestrial Food Chain
Source: PLoS One. 2013 Nov 4;8(11):e78444. doi: 10.1371/journal.pone.0078444 (PMC3817214; doi:10.1371/journal.pone.0078444)
Supplement: Table S6 — General Linear Model results of relationships between soil, leaf, acorn or weevil Mg and climate variables (MAP = mean annual precipitation, MAT = mean annual temperature). “ns” indicates no significant relationship. (DOCX) [file pone.0078444.s009.docx]

**Table S6** General Linear Model results of relationships between soil, leaf, acorn or weevil Mg and climate variables (MAP = mean annual precipitation, MAT=mean annual temperature). “ns” indicates no significant relationship.

| Mg | n | Model (r^2^) | | |
| --- | --- | --- | --- | --- |
|  |  | MAP | MAT | MAP*MAT |
| Soil | 35 | 0.457 | 0.131 | 0.452 |
| Leaf | 36 | 0.168 | 0.175 | 0.189 |
| Acorn | 21 | 0.224 | 0.289 | 0.273 |
| Weevil | 20 | ns | 0.353 | 0.348 |
